# Intention to Adopt mHealth Apps Among Informal Caregivers: Cross-Sectional Study

**DOI:** 10.2196/24755

**Published:** 2021-03-17

**Authors:** Fereshteh Ghahramani, Jingguo Wang

**Affiliations:** 1 College of Computing and Digital Media DePaul University Chicago, IL United States; 2 Department of Information Systems and Operations Management University of Texas at Arlington Arlington, TX United States

**Keywords:** mobile health, cross-sectional study, informal caregivers, mobile app, caregiving app, mobile phone

## Abstract

**Background:**

Caregiving responsibility can change caregivers’ lives; modify their emotions; and make them feel frustrated, fearful, and nervous, thereby imposing physical and mental stress. Caregiving-related mobile apps provide a platform for obtaining valuable and trusted information, connecting more easily with other caregivers, monitoring medications, and managing appointments, and assessing health requirements and conditions of care receivers. Such apps also incorporate valuable resources that address care for the caregivers. Despite the potential benefits of caregiving-related apps, only a limited number of caregivers have adopted and used them.

**Objective:**

The aim of this study is to explore the important factors that affect caregivers’ intentions to integrate related mobile apps into their routine caregiving responsibilities.

**Methods:**

Using the protection motivation theory, we conducted a cross-sectional study among 249 participants. Purposive sampling was used to target participants who met 4 inclusion criteria: US residents, owning and using a smartphone, informal caregivers (individuals who give care to a friend or family member without payment) who provided at least 8 hours of care per week in the past year, and those currently not using any mobile app for caregiving purposes. We created a survey using Qualtrics and posted it on Amazon’s Mechanical Turk website. Participants received monetary compensation after successful completion of the survey.

**Results:**

We found that capabilities and skills of caregivers to use mobile apps, the app’s effectiveness in responding to the needs of caregivers, the degree of control of caregivers over their responsibilities, and the decisions they make for their care receivers can predict their willingness to adopt caregiving-related apps. In addition, the severity of health status and vulnerability of care receivers to unexpected health changes indirectly shape their caregivers’ decisions to adopt and use mobile apps for caregiving purposes.

**Conclusions:**

This study explores the important factors that affect informal caregivers’ intentions to adopt related mobile apps into their routine caregiving responsibilities. The results contribute to both mobile health adoption and the caregiving literature, and they offer significant implications for developers, health care practitioners, and policy makers.

## Introduction

### Background

In 2017, only approximately 37 million patients were admitted to US hospitals [1]. As the number of individuals needing hospitalization has increased in recent years [2], hospitals have mainly limited their beds to acute care and left the rest of treatments in the form of outpatient settings to the patients’ loved ones [3,4]. Unpaid family or informal caregivers are, therefore, the backbone of the long-term care system and offer the majority of long-term care that patients require in the United States [5]. In addition, informal caregiving is essential for sustaining adults with disabilities or chronic health conditions [6].

Caregiving responsibilities primarily include providing informational and emotional support, dealing with financial concerns, and managing medical care [4]. These responsibilities also consist of more basic support, such as assistance in eating, bathing, grocery shopping, and meal preparation [7]. Many informal caregivers experience positive feelings such as satisfaction, a sense of gratification for giving back to those who cared for them, and improved family relationships [8,9]. However, the nature and amount of such responsibilities may change caregivers’ lives; modify their emotions; and make them feel frustrated, fearful, and nervous, all resulting in a decline in their quality of life [10].

Estimates of the number of informal caregivers in the United States vary widely. According to the American Association of Retired Persons, there are currently more than 40 million unpaid caregivers in the United States [11], about 7 potential informal caregivers per adult [12]. Although studies indicate that the number of informal caregivers will continue to rise, it is expected that the number of individuals who need care will be far from the number of those who offer that care in the near future (about 4 potential informal caregivers per adult) [12,13]. There are 2 main reasons for this result. First, the number of individuals who will be over 65 years old by 2050 is expected to be 2 times more than that number in 2010; the majority of those individuals will be dealing with various chronic conditions and a decline in quality of life [14,15]. Second, the traditional American family structure has experienced fundamental changes in recent years. Family sizes continue to decrease as a result of higher rates of those who have never married or are divorced or are affected by infertility and childlessness. In addition, there is a higher chance that women, who are the backbone of informal caregiving, are in the workforce [16,17].

This shrinkage in the number of potential informal caregivers per adult increases the amount of responsibility and inflicts more physical and mental stress among the existing caregivers [14]. The ubiquity of mobile technology and its applications has the potential to reduce such stress. In general, mobile apps are reasonably priced and user friendly and offer an information repository collected from various sources [18-20]. Caregiving-related apps are specifically designed to provide users with a platform to gain appropriate and trusted information, manage medication taking, improve communication with care providers and support groups, connect with counterparts, reserve transportation, and manage the health condition of care receivers in an organized manner [21].

Although there are hardly any studies in the literature that investigate the role of caregiving-related apps in reducing caregiving-related stress [22], it is very likely that such apps significantly lessen the stress caregivers face by providing a convenient platform to receive informational and emotional support [14]. Despite the considerable role of caregiving apps in reducing stress and improving the overall quality of life among caregivers and although more than 57% of American caregivers have a smartphone, only 40% of them use a caregiving-related app [23]. This raises the concern of finding solutions to increase caregivers’ access to and effective use of such beneficial resources.

Some studies highlight the roles of caregivers’ digital literacy and sociodemographic factors on their natural propensity to use various internet-based tools and services for caregiving purposes in general [24,25]. However, the current understanding of caregivers’ intentions to use related mobile apps for their responsibilities is limited, and we could not find any published studies that directly investigated the influential factors.

As such, our objective is to provide insights into this issue. To explore the important factors that affect their intentions to integrate mobile apps into their routine caregiving responsibilities, we designed a cross-sectional study using the protection motivation theory (PMT) perspective [26]. PMT is among the most influential explanatory theories in the literature to predict an individual’s intention to adopt recommended actions (adoption of related mobile apps in this study) [27]. It has also been empirically applied to both technological and nontechnological solutions [28].

### Theoretical Background

PMT suggests 2 consecutive appraisals that explain the process whereby individuals adopt a recommended action: threat and coping [29]. If an individual is exposed to a stressful or fearful situation, a personal perception of threat arises. If the threat is perceived to be appropriate and possibly harmful, coping appraisal will occur [28].

**Figure 1:**
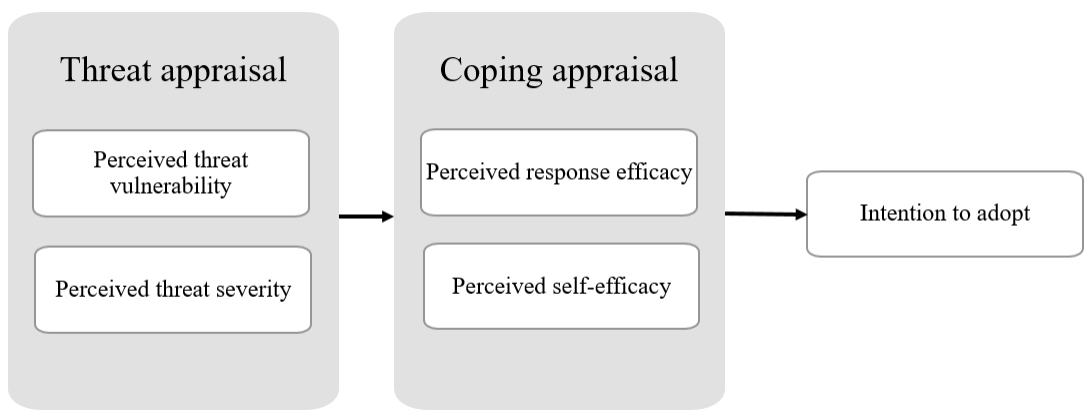
Protection motivation theory model.

On the basis of the threatening event, individuals first assess the level of danger in 2 aspects: (1) perceived threat severity, which is the individual’s assessment of the seriousness of the threat, and (2) perceived threat vulnerability, which is the individual’s assessment of the likelihood of coming across the threat personally. Once the threat has been assessed, individuals assess their ability to cope with the threat and form their perceptions of the efficacy of the recommended action in 2 aspects: (1) self-efficacy, which is the individuals’ assessment of their capability to perform the recommended action, and (2) response efficacy, which is the individual’s assessment of the efficacy and benefits of the recommended action [26,29,30]. This efficacy assessment affects individuals’ intentions to adopt the recommended action. Figure 1 illustrates the PMT model.

### Research Model and Hypotheses

#### Overview

Informal caregivers experience a vast amount of uncertainty and psychological distress [31] because of the difficulty in predicting their care receivers’ disease and care progress [32]. Adopting and using mobile health apps can be considered a recommended action to reduce caregivers’ stress. Building on PMT, we propose that, reflecting care receivers’ physical and mental condition, caregivers form perceptions about the severity and vulnerability of their care receivers’ health threats (threat appraisal). If they perceive a significant and harmful degree of threat, caregivers will assess both their responses and self-efficacy to cope with the situation (coping appraisal). Caregivers who expect that mobile apps can help them (high response efficacy) and have the efficacy to operate the technologies (high self-efficacy) are expected to begin using related mobile apps for caregiving purposes [28].

**Figure 2:**
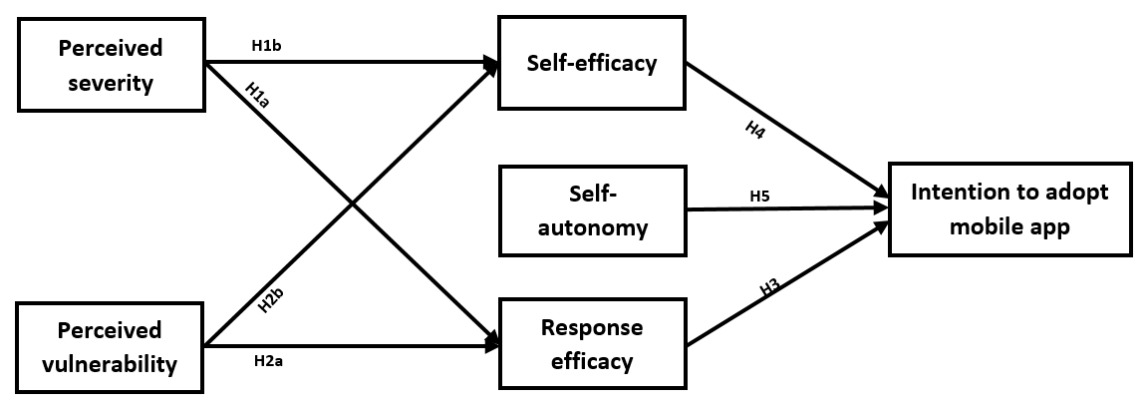
Research model.

Moreover, as self-efficacy only reflects caregivers’ perceptions of their general capability to use an app [33], we also need to consider the degree to which caregivers have control over their caregiving responsibilities and the decisions they make for their care receivers. This is called perceived self-autonomy, and previous studies have verified it as a major contributor to technology acceptance together with self-efficacy [34,35]. When caregivers feel autonomous in choosing a mobile app, they perceive it as useful and easy to adopt [34]. Thus, we propose that perceived self-autonomy is an antecedent that influences caregivers’ intention to adopt and use caregiving-related apps. The proposed research model is illustrated in Figure 2. The construct definitions are presented in Table 1.

**Table 1:** Definitions of constructs.

Construct	Definition
Perceived threat severity	Degree to which a caregiver assesses the seriousness of their care receiver’s health status
Perceived threat vulnerability	Degree to which a caregiver assesses the susceptibility of their care receiver to a sudden change in health status or an unexpected health condition
Perceived self-efficacy	Degree to which a caregiver believes that they are capable and have the necessary skills to use a caregiving-related mobile app
Perceived response efficacy	Degree to which a caregiver believes that a caregiving-related mobile app will effectively prevent threats related to their care receiver’s health condition
Perceived self-autonomy	Degree to which a caregiver has control over caregiving responsibilities and the decision they make for the care receiver
Intention to adopt	Caregiver’s willingness to adopt a caregiving-related app

#### Threat Appraisal

Depending on the physical and mental conditions of the care receiver, the caregivers assesses the seriousness of the care receiver’s health status. This assessment can influence the significance of caregiving-related apps by manipulating both responses and self-efficacy perceptions of caregivers [28]. More specifically, as a caregiver feels that their care receiver deals with a more severe health situation, he or she is more willing to trust sources that can provide the support he or she is looking for to help him or her cope with the situation.

Therefore, the caregivers perceives the app as a more capable and qualified tool to effectively help him or her address the threat in a way that he or she might not have previously recognized. It can also persuade caregivers to re-examine their abilities to use the recommended app for caregiving activities. As the care receiver’s health status is perceived to be more severe, the caregivers will rely more on him or her and feel more confident about using a caregiving-related app as one of the few means to deal with the severe situation. Therefore, we hypothesize the following:

Hypothesis 1a (H1a): The caregiver’s perception of the care receiver’s severity of health status positively influences the caregiver’s perception of the mobile app’s response efficacy.

Hypothesis 1b (H1b): The caregiver’s perception of the care receiver’s severity of health status positively influences the caregiver’s perception of his or her self-efficacy in using the mobile app.

Similar to the logic of perceived severity, when caregivers notice that there is a likelihood that their care receiver will encounter a sudden and unanticipated change in their health condition, they tend to perceive the mobile app as comprehensive and effective enough to offer them the support the caregivers are looking for during this time.

Therefore, it is expected that as the threat of facing unexpected health changes becomes more probable, caregivers will perceive the mobile app as a more effective and efficient tool to help them in their caregiving responsibilities. In addition, caregivers will find themselves more capable and confident in using the mobile app as a tool to lessen the burden and the effects of the vulnerable situation. So, we proposed the following hypothesis:

Hypothesis 2a (H2a): The caregiver’s perception of the care receiver’s vulnerability to unexpected health changes positively influences the caregiver’s perception of the mobile app’s response efficacy.

Hypothesis 2b (H2b): The caregiver’s perception of the care receiver’s vulnerability to unexpected health changes positively influences the caregiver’s perception of his or her self-efficacy in using the mobile app.

#### Coping Appraisal

Perceived response efficacy is a cognitive process by which caregivers develop thoughts about the effectiveness and capability of the app to address their needs while they are dealing with a threatening situation [26,29]. In other words, the caregivers’ perception of the mobile app’s response efficacy determines whether they choose that app to help them handle the threat or not [36]. According to PMT, a high level of response efficacy forms a positive disposition toward the recommended solution. So, we proposed the following hypothesis:

Hypothesis 3 (H3): The perceived response efficacy of the caregiving-related app has a positive effect on the caregiver’s intention to adopt the app.

Even if a caregiver believes that the app is effective enough to address their needs, he or she still needs to consider their own ability to successfully install and effectively use the app [28]. Caregivers who perceive themselves as capable of using the app are more willing to adopt and use such apps to address their caregiving-related requirements [37]. Therefore, we proposed the following hypothesis:

Hypothesis 4 (H4): The caregiver’s perceived self-efficacy has a positive effect on his or her intention to adopt the caregiving-related app.

#### Self-Autonomy

Furthermore, if caregivers have some freedom of action in their caregiving activities and have a say regarding what mobile apps they want to use, there is a higher chance that they will become intrinsically motivated or maintain the primary levels of intrinsic motivation to adopt and use the app [38]. Such a feeling of control can also enhance caregivers’ positive feelings toward using the app [39,40]. Therefore, we proposed the following hypothesis:

Hypothesis 5 (H5): The caregiver’s perceived self-autonomy has a positive effect on his or her intention to adopt the caregiving-related app.

## Methods

### Recruitment

To test the proposed model, we recruited participants through Amazon’s Mechanical Turk, which is a web-based crowdsourcing market for registered users to participate in various tasks and receive a predetermined amount of money upon successful completion [41]. Studies in the domain of information systems have confirmed the validity and reliability of the results [42,43]. Purposive sampling was used to target participants who met the following inclusion criteria [44]: (1) US residents, (2) owning and using a smartphone, (3) informal caregivers (individuals who give care to a friend or family member without payment) who provided at least 8 hours of care per week in the past year, and (4) currently not using any mobile app for caregiving purposes. We obtained approval from the Institutional Review Board for the explained approach.

### Instrument Development

We adapted measures from a set of empirically validated studies in the literature and used multi-item measures to enhance the validity and reliability of the measurement. All the main constructs were reflective. For the constructs drawn from PMT, measurement items were adapted from Witte [45] and modified to fit the context of our study. Under threat appraisal, 5 items (mean 3.76, SD 0.74) and 4 items (mean 4.03, SD 0.69) were used to assess perceived vulnerability (eg, “My care-receiver is at risk for getting health threats”) and perceived severity (eg, “I believe that threats to my care receiver's health are severe”), respectively. For coping appraisal, 6 items (mean 3.37, SD 0.86) assessed response efficacy (eg, “Using mobile apps is effective in monitoring my care-receiver’s health condition remotely”), and 4 items (mean 3.79, SD 0.78) were used to measure self-efficacy (eg, “I feel confident using mobile health applications for my caregiving activities”). Intention to use items was adapted from a widely used scale in the literature [46] (3 items; mean 2.98, SD 0.93) and changed appropriately to fit our study (eg, “I plan to use mobile apps to manage my care-receiver’s health status in the next 3 months”). Items for perceived self-autonomy were adapted from the study by Deci et al [47] (5 items; mean 3.89, SD 0.83) and altered to suit our context (eg, “I have some choice in what I want to do in my caregiving activities”). For all the aforementioned constructs, participants indicated their level of agreement with each item using a 5-point Likert-type scale ranging from *strongly disagree* (1) to s*trongly agree* (5).

Questions were randomly shown to the participants to minimize the order-effect bias. Two experts reviewed the initial questionnaire to ensure face validity. We revised the survey based on their comments and feedback. We also conducted a pilot study among master’s students at a large university in the southwestern region of the United States and made appropriate changes to the survey based on the results. Previous literature, pilot tests, and numerous series of pretests allowed us to confirm the content validity of our instrument and measures [48]. The final measurement items are listed in Table 2. The Checklist for Reporting Results of Internet E-Survey is available in Multimedia Appendix 1 [49].

**Table 2:** Instrument items.

Construct			Items		References
**Dependent variable**					
	Intention to use	It is my intention to use mobile applications in caregiving activities. I plan to use mobile apps to manage my care-receiver’s health status in the next 3 months. I am likely to learn about using mobile apps in caregiving activities.		Venkatesh et al [46]	
**Threat appraisal**					
	Vulnerability	My care-receiver can be subjected to a sudden change in health condition. My care-receiver is at risk for getting health threats. It is possible that my care-receiver will contract health threats. It is likely that my care-receiver requires an urgent care. It is likely that my care-receiver will contract health threats.		Witte [45]	
	Severity	If my care-receiver faces an unexpected health problem, it would be serious. I believe that threats to my care-receiver’s health are severe. I believe that threats to my care-receiver’s health are serious. I believe that threats to my care-receiver’s health are significant.		Witte [45]	
**Coping appraisal**					
	Response efficacy	Mobile apps will help me manage medication for my care-receiver. Mobile apps serve as an effective disease reference and caregiving adviser. Mobile apps enable me to keep a log of medical information for my care-receiver. Mobile apps work in preventing health threats due to mismanagement of medications. Using mobile apps is effective in monitoring my care-receiver’s health condition remotely (eg, heart rate, oxygen level, or other vital signs). If I use mobile apps in my caregiving activities, my care-receiver is less likely to get health threats due to mismanagement of medications.		Witte [45]	
	Self-efficacy	I feel confident using mobile health applications for my caregiving activities. I am able to use mobile apps. Mobile apps are easy to use. Using mobile apps is convenient.		Witte [45]	
	Perceived self-autonomy	In my caregiving activities, I can decide which mobile apps I want to use. In my caregiving activities, I have a say regarding what mobile apps I want to use. I feel that I will use mobile apps for caregiving purposes because I want to. I feel a certain freedom of action in my caregiving activities. I have some choice in what I want to do in my caregiving activities.		Deci et al [47]	

### Sample Characteristics

A total of 249 valid responses were collected. The average time that our responders provided care for their current care receivers and for various individuals in general was 50 and 83 months, respectively. A total of 32.5% (81/249) of the care receivers were financially dependent on their caregivers. Tables 3 and 4 summarize the key demographic variables of the respondents and their care receivers.

**Table 3:** Key demographics of care receivers.

Variable			Percentage, n (%)
**Age (years)**			
	≤18	28 (11.2)	
	18-49	39 (15.7)	
	50-69	55 (22.1)	
	≥70	127 (51.0)	
**Education**			
	High school or general educational development	106 (42.6)	
	Some college or bachelor’s degree	89 (35.7)	
	Master’s degree	32 (12.9)	
	Professional degree	17 (6.8)	
	Doctoral degree	5 (2.0)	
**Race**			
	White	185 (74.3)	
	African American	27 (10.8)	
	Hispanic	22 (8.9)	
	Asian	11 (4.4)	
	Native American	2 (0.8)	
	Pacific Islander	2 (0.8)	
**Gender**			
	Male	102 (41.0)	
	Female	147 (59.0)	
**Relationship with care receiver**			
	Parents	46 (18.5)	
	Friend	132 (53.0)	
	Family friend	25 (10.0)	
	Spouse	28 (11.3)	
	Child	18 (7.2)	
**Reason for receiving care**			
	Any form of disease	78 (31.3)	
	Old age	77 (30.9)	
	Disability	59 (23.7)	
	Mental disorder	35 (14.1)	

**Table 4:** Key demographics of caregivers (N=249).

Variable			Percentage, n (%)
**Age (years)**			
	≤18	0 (0)	
	18-24	33 (13.3)	
	25-34	88 (35.3)	
	35-44	64 (25.7)	
	45-54	35 (14.1)	
	55-64	21 (8.4)	
	≥65	8 (3.2)	
**Education**			
	High school or general educational development	22 (8.8)	
	Some college or bachelor’s degree	181 (72.7)	
	Master’s degree	42 (16.9)	
	Professional degree	1 (0.4)	
	Doctoral degree	3 (1.2)	
**Race**			
	White	193 (77.5)	
	African American	24 (9.7)	
	Hispanic	16 (6.4)	
	Asian	9 (3.6)	
	Native American	2 (0.8)	
	Pacific Islander	5 (2.0)	
**Gender**			
	Male	78 (31.3)	
	Female	171 (68.7)	
**Marital status**			
	Single without children	72 (28.9)	
	Single with children	33 (13.3)	
	Married without children	18 (7.2)	
	Married with children	98 (39.4)	
	Life partner without children	13 (5.2)	
	Life partner with children	15 (6.0)	
**Income, US ($)**			
	≤20,000	47 (18.9)	
	20,000-40,000	72 (28.9)	
	40,000-60,000	61 (24.5)	
	60,000-80,000	41 (16.4)	
	≥80,000	28 (11.2)	

### Data Analysis

A partial least squares approach was used to test the proposed model and associated hypotheses. We used the SmartPLS software package (version 3.2.6, SmartPLS GmbH) to analyze the data [50]. To estimate the path coefficient weights and their significance, we used bootstrapping procedures with 5000 resamples.

To assess the measurement model, we conducted a confirmatory factor analysis. The results are summarized in Table 5. All of the latent constructs are modelled to be reflective. For each latent construct, the path loadings, *t* statistics, and SE were calculated. At α=.05, all the measures’ path loadings are significant with a value of more than 0.7 [51], indicating that more than 50% of the variance is shared between each construct’s items [52].

**Table 5:** Confirmatory factor analysis results.

Construct and items		Loading	*t* test	SE	Average variance extracted		Composite reliability		Cronbach α	
**Intention to use**						0.810		0.927		.882
	Int^a^1	0.911	61.307	0.015						
	Int2	0.930	107.161	0.009						
	Int3	0.857	33.746	0.025						
**Vulnerability**						0.656		0.905		.869
	Vul^b^1	0.750	10.763	0.070						
	Vul2	0.834	11.179	0.075						
	Vul3	0.818	9.688	0.084						
	Vul4	0.767	13.247	0.058						
	Vul5	0.873	17.901	0.049						
**Severity**						0.758		0.926		.895
	Sev^c^1	0.861	18.791	0.046						
	Sev2	0.835	13.664	0.061						
	Sev3	0.897	23.558	0.038						
	Sev4	0.888	23.897	0.037						
**Response efficacy**						0.681		0.927		.906
	RE^d^1	0.874	63.174	0.014						
	RE2	0.843	33.026	0.026						
	RE3	0.842	36.862	0.023						
	RE4	0.846	25.670	0.033						
	RE5	0.794	20.271	0.039						
	RE6	0.745	17.234	0.043						
**Self-efficacy**						0.636		0.874		.813
	SE^e^1	0.792	30.109	0.026						
	SE2	0.745	13.692	0.054						
	SE3	0.787	11.989	0.066						
	SE4	0.862	31.175	0.028						
**Self-autonomy**						0.615		0.888		.853
	SA^f^1	0.837	28.530	0.029						
	SA2	0.818	22.784	0.036						
	SA3	0.833	43.420	0.019						
	SA4	0.714	10.462	0.068						
	SA5	0.708	9.575	0.074						

^a^Int: intention to use.

^b^Vul: vulnerability.

^c^Sev: severity.

^d^RE: response efficacy.

^e^SE: self-efficacy.

^f^SA: self-autonomy.

To examine convergent validity, both composite reliability and average variance extracted (AVE) were obtained [53]. All constructs met the acceptable score of 0.5 for AVE, confirming their reliability [54]. Moreover, the composite reliability values were between 0.874 and 0.927.

The square root of the AVE was also used to verify discriminant validity [54]. The results are presented in Table 6. For each latent construct, the square root of the AVE was higher than all its cross-correlations. Moreover, higher values of interconstruct correlations confirmed the greater variance among each construct’s specific measures compared with other measures [55]. All things considered, we can confirm that all the indicators were valid through their constructs.

As data for all the variables were collected in a single survey, common method variance (CMV) could have an excessive effect on the results [56]. Harman’s single factor test was conducted [57] to find out the extent of this effect. According to this approach, with a factor analysis, if only a single factor arises, or a factor explains the majority of variance among all the measures, we can conclude that CMV is a significant issue in the sample [56]. After conducting an exploratory factor analysis among all the items, 6 factors were obtained that explained more than 70% of the variance. All the extracted factors had an eigenvalue >1, and the highest factor accounted for only 33% of the variance. This indicates that CMV is not a serious concern in this data set.

**Table 6:** Matrix of latent constructs’ correlations.

Construct	Mean (SD)	Construct 1	Construct 2	Construct 3	Construct 4	Construct 5	Construct 6
1. Intention to use	2.976 (0.927)	*0.900* ^a^	–^b^	–	–	–	–
2. Vulnerability	3.764 (0.736)	0.609	*0.810* ^a^	–	–	–	–
3. Severity	4.034 (0.691)	0.129	0.646	*0.871* ^a^	–	–	–
4. Response efficacy	3.371 (0.858)	0.607	0.245	0.201	*0.825* ^a^	–	–
5. Self-efficacy	3.790 (0.780)	0.479	0.111	0.208	0.652	*0.797* ^a^	–
6. Self-autonomy	3.892 (0.829)	0.472	0.211	0.257	0.561	0.631	*0.784* ^a^

^a^Italicized values in the diagonal row are the square root of the average variance extracted.

^b^not applicable.

## Results

**Figure 3:**
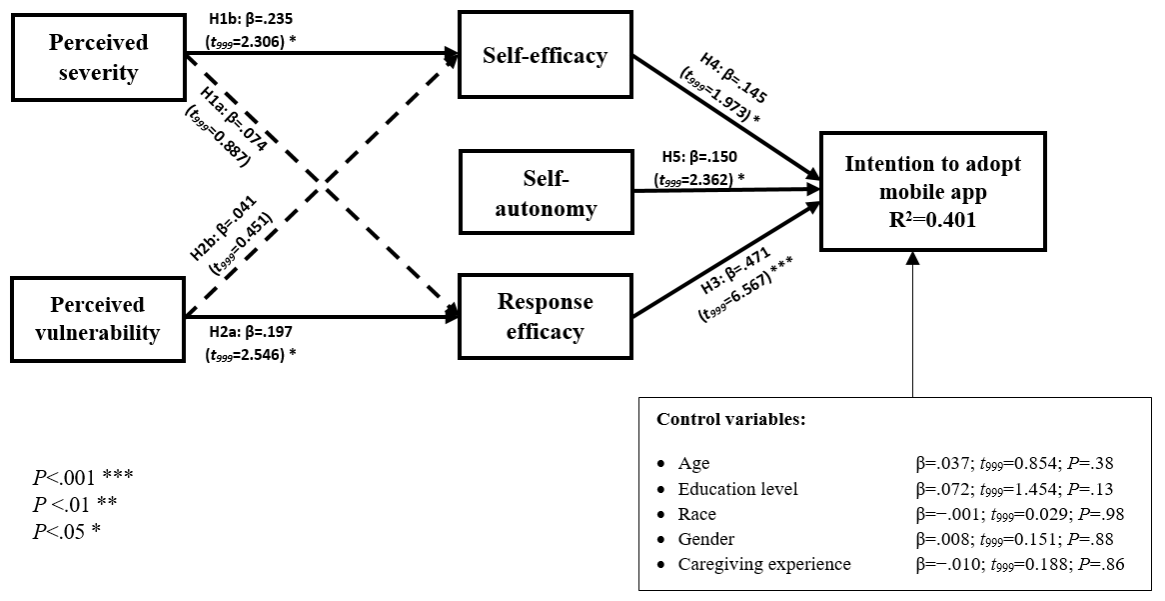
Partial least squares results.

The results of data analysis, including *R*^2^ values, standardized path coefficients, associated *t* values, and path significance, are depicted in Figure 3. To test the significance of the path coefficients in the structural model, we used a bootstrapping approach with 1000 resamples.

The *R*^2^ value of intention to adopt a caregiving mobile app is 0.401, indicating that more than 40% of the variance in intention to adopt can be explained by response efficacy, self-efficacy, and self-autonomy. As this amount of variance is more than 10%, we can claim that the proposed model is valid and acceptable [58].

The results indicate that response efficacy (*β*=.471; *t_999_*=6.567; *P*<.001), self-efficacy (*β*=.145; *t_999_*=1.973; *P*=.049), and self-autonomy (*β*=.150; *t_999_*=2.362; *P*=.03) had significant effects on the intention to adopt, supporting H3, H4, and H5. Moreover, perceived vulnerability and perceived severity had significant effects on response efficacy (*β*=.197; *t_999_*=2.546; *P*=.008) and self-efficacy (*β*=.235; *t_999_*=2.306; *P*=.01), respectively, supporting H2a and H1b. Perceived vulnerability and perceived severity, however, do not significantly affect self-efficacy (*β*=.041; *t_999_*=0.451; *P*=.63) and response efficacy (*β*=.074; *t_999_*=0.887; *P*=.35), respectively. Therefore, we do not have sufficient evidence to support H2b and H1a. The results are summarized in Table 7.

To account for individual differences in the proposed model, we controlled for the effects of the caregivers’ age, education level, race, gender [46,58], and caregiving experience. None of these variables had a significant effect on the intention to adopt a caregiving mobile app.

**Table 7:** Summary of results.

Hypothesis	Result
H1a. The caregiver’s perception of the care receiver’s severity of health status positively influences the caregiver’s perception of the mobile app’s response efficacy.	Not supported
H1b. The caregiver’s perception of the care receiver’s severity of health status positively influences the caregiver’s perception of his or her self-efficacy to use the mobile app.	Supported
H2a. The caregiver’s perception of the care receiver’s vulnerability to unexpected health changes positively influences the caregiver’s perception of the mobile app’s response efficacy.	Supported
H2b. The caregiver’s perception of the care receiver’s vulnerability to unexpected health changes positively influences the caregiver’s perception of his or her self-efficacy to use the mobile app.	Not supported
H3. The perceived response efficacy of the caregiving-related app has a positive effect on the caregiver’s intention to adopt the app.	Supported
H4. The caregiver’s perceived self-efficacy has a positive effect on his or her intention to adopt the caregiving-related app.	Supported
H5. The caregiver’s perceived self-autonomy has a positive effect on his or her intention to adopt the caregiving-related app.	Supported

## Discussion

### Principal Findings

The primary goal of this study is to investigate the factors that affect caregivers’ intentions to adopt caregiving-related mobile apps based on a model that contextualizes PMT. The results indicate support for the proposed model with decent explanatory power.

As the analyses illustrate, caregivers’ capabilities and skills to use mobile apps and the app’s effectiveness in responding to caregivers’ needs can predict their willingness to adopt related apps. In addition, our results indicate that the degree of control of caregivers over their responsibilities and the decisions they make for their care receivers can also increase the likelihood of adopting such mobile apps. Interestingly, the app’s effectiveness in responding to caregivers’ needs had the strongest effect on their intention to adopt such apps.

We also found that as the care receiver’s health status is perceived to be more severe, caregivers will count more on their capability to use a caregiving-related app. In addition, as the threat of facing unexpected health changes becomes more likely, caregivers will perceive the mobile app as a more efficient tool to help them with their responsibilities. These findings are consistent with previous studies on PMT [28].

However, we did not find enough evidence to support the effect of the care receiver’s severity of health status on the caregiver’s perception of the mobile app’s effectiveness. In addition, the results indicate that the effect of the care receiver’s vulnerability to unexpected health changes on the caregiver’s perception of their self-efficacy in using the mobile app is not significant. Although these results are not congruent with PMT, there are various other studies that confirm that individuals, in most cases, believe that threats either happen only to others or influence other individuals more than themselves [59]. This belief helps them maintain a sense of invulnerability and explains why we did not find evidence to support 2 of the hypotheses.

### Implications

As mentioned earlier, caregiving apps play a considerable role in reducing stress and improving the overall quality of life of informal caregivers. Therefore, it is important to devote time, money, and effort to develop and promote caregiving-related apps to enhance both caregivers’ and care receivers’ well-being. In this regard, the results of this study provide several practical implications for developers, health care practitioners, and policy makers.

#### Developers

Currently, more than 318,000 mobile health apps are available to consumers in the top app stores globally, and more than 200 apps are being added every day [60]. However, only a small number of these apps are designed specifically to help caregivers with the challenges they face because of their responsibilities (excluding apps for professional caregiving organizations or those that help in locating such services and organizations) [14]. Developing more caregiving-related apps gives caregivers the opportunity to have several options to choose from and enhances their sense of control over a stressful situation.

Moreover, caregiving-related apps must offer a comprehensive set of features that caregivers typically look for. Although previous studies have confirmed the significant and positive effects of caregivers’ quality of life on the patients’ quality of life [61], only 20% of current caregiving-related apps have a collective set of features required by caregivers at different stages of their responsibilities [14]. According to our results, response efficacy has the strongest effect on behavioral intention. Including a broad range of features that address care for the care receiver (ie, appointment and medication management and reliable information about the disease or specific situation) can increase the chance of the caregivers’ adoption of those apps.

In addition to the resources and features on how to manage their care receivers’ condition, incorporating features that address care for the caregivers can help them find a way to meet their needs in one place. This can increase the effectiveness of mobile apps [62] and possibly increase the likelihood of adopting these apps. For instance, apps may contain features to (1) learn and assess emotions such as journaling as a tool to keep track of moods and mood shifts; (2) manage and reduce personal stress through quick meditative activities and breathing exercises that fit into busy routines or through the use of in-app coloring books; (3) receive emotional and social support such as app-based chats and support groups [14]; and (4) receive informational support such as podcasts or discussions on caregivers’ self-care, including suggestions, resources, and inspiring words from others in the community of caregivers.

Finally, based on our results, caregiving-related apps should be designed in a user-friendly, straightforward way to help caregivers locate services more easily, considering the time constraints of caregivers. Moreover, nearly 55% of caregivers are aged 50 years or more [63]. This makes it more important to design such apps in a simple and easy-to-operate manner to ensure that both younger and older adults can equally benefit from those apps.

#### Health Care Practitioners

Lack of awareness about the appropriate options or possible benefits, feeling overwhelmed by the available choices, and shortness of time to conduct appropriate research are among the biggest barriers of caregivers’ adoption of technology [64]. By educating physicians, nurses, social workers, and personal care aides on the available caregiving apps and asking them to spread the word by suggesting approved ones, there is a higher chance that those apps will find their way into caregivers’ routines.

Moreover, based on our results, awareness of the care receiver’s health condition and how it may progress can affect the perceived effectiveness and efficiency of the suggested app among caregivers. Rather than only suggesting the app, it would be better to suggest why and how (based on the severity of the care receiver’s health status and vulnerability to unexpected health changes) the suggested app can take some weight off of caregivers’ shoulders. This indirectly increases the chances of adopting such apps.

Flyers and signage are also great tools to target caregivers and to let them know the names and features of available caregiving-related apps in the market. Information about the variety of services offered by the apps, assurance of consistency with federal or state security and privacy acts, and instructions on ways to find and download the app (ie, relevant screenshots and licensed QR codes) are examples of the essential information that should be included in such flyers.

#### Policy Makers

Besides spreading the word to the worlds of developers and health practitioners, implementing various incentives might be a good motivator to increase adoption rates among caregivers. Insurance providers and policy makers can take into account policies to promote the adoption and use of caregiving-related apps [65]. Covering the cost of caregiving-related app purchases by insurance companies, reducing the price of apps or in-app purchases, raising research funds to develop more apps in this category, setting standards to require a minimum level of quality for those apps, and supporting young and motivated developers who are interested in this area are only a few examples of systematic changes that increase the likelihood of adopting and using caregiving-related apps [66].

### Limitations and Future Research

This study has some limitations. First, the survey was limited to US caregivers, raising the external validity issue. Future research is required to generalize the results and offer an inclusive perspective of caregivers’ adoption of related mobile apps in the context of other countries. Second, the collected information and derived results were based on self-reported data. This can increase the likelihood of some biases such as social desirability. Further studies may use a more inclusive data collection technique to measure adoption more rigorously. Finally, there is still some unexplained variance in the intention to adopt mobile apps. Although including more constructs may have improved the explanatory factor of the proposed model, we intended to maintain a parsimonious extension of PMT [28]. Additional studies are required to investigate other effective factors.

### Conclusions

Grounded on PMT, this study investigates the factors that affect the intentions of informal caregivers to adopt related mobile apps for their routine caregiving responsibilities. The results of the survey of 249 US-based informal caregivers indicated that caregivers’ degree of control over their responsibilities and choices, their perception of the app’s effectiveness in responding to their needs, and their capability to use mobile apps play a positive role in their willingness to adopt caregiving-related apps. Furthermore, care receivers’ vulnerability to unpredicted health changes and the severity of their health status indirectly affect their caregivers’ intentions to adopt such mobile apps. These findings offer important contributions to the field and have significant implications for developers, health care practitioners, and policy makers.

## Appendix

Multimedia Appendix 1 The checklist for reporting results of our internet e-survey (CHERRIES).

## Abbreviations

AVE: average variance extracted
CMV: common method variance
PMT: protection motivation theory
