# Supplementary material for: Intention to Adopt mHealth Apps Among Informal Caregivers: Cross-Sectional Study
Source: JMIR Mhealth Uhealth. 2021 Mar 17;9(3):e24755. doi: 10.2196/24755 (PMC8294641; doi:10.2196/24755)
Supplement: Multimedia Appendix 1 [file mhealth_v9i3e24755_app1.docx]

**Appendix**

| **Item Category** | **Checklist Item** | **Explanation** |
| --- | --- | --- |
| Design | Describe survey design | Target population: participants that met the following criteria: (1) U.S. resident, (2) own and use a smartphone, (3) informal caregiver (we provided the definition) who provided at least eight hours of care per week in the past year, and (4) currently not using any mobile application for caregiving purposes.  The sample is a convenience sample. |
| IRB (Institutional Review Board) approval and informed consent process | IRB approval | The University of Texas at Arlington IRB reviewed and approved this study. |
|  | Informed consent | Survey was created in Qualtrics and posted on Amazon’s Mechanical Turk (MTurk) website. Registered users who clicked on the survey were directed to the welcome page. In this page, we described the investigators, purpose, and length of this study. We also explained our plan to collect and store data. Those who agreed to participate were taken to the filtering questions’ page to check whether they met the four inclusion criteria. |
|  | Data protection | We did not collect any personal identifiable information. Moreover, the survey and collected data were stored on a secure server. |
| Development and pre-testing | Development and testing | We adapted survey measures from a set of empirically-validated studies in the literature and used multi-item measures to enhance the validity and reliability of measurement. Two experts reviewed the initial questionnaire to ensure face validity. We revised the survey based on their comments and feedback. We also conducted a pilot study among master’s students at a large university in the south-western region of the U.S. and made appropriate changes to the survey based on the results. |
| Recruitment process and description of the sample having access to the questionnaire | Open survey versus closed survey | Open survey. |
|  | Contact mode | We did not contact any participant outside of the MTurk platform. |
|  | Advertising the survey | The survey was only posted on MTurk website. No other advertising was done. |
| Survey administration | Web/E-mail | Web survey. |
|  | Context | MTurk is an online crowdsourcing market for registered users to participate in various tasks and receive a predetermined amount of money upon successful completion. Users need to agree to the Amazon Mechanical Turk Participation Agreement to be able to create an account. |
|  | Mandatory/voluntary | Voluntary |
|  | Incentives | Monetary incentives were offered. |
|  | Time/Date | Data were collected September 2017 |
|  | Randomization of items or questionnaires | Questions were randomly shown to participants to minimize the order-effect bias. |
|  | Adaptive questioning | We did not use adaptive questioning. |
|  | Number of Items | 3 to 4 questions per page. |
|  | Number of screens (pages) | 5 pages |
|  | Completeness check | Each question needed a response to advance the participant to the next page. We provided a “not applicable” option for all the questions.  We conducted the completeness check while cleaning the data on the analysis phase. |
|  | Review step | Respondents were able to review and change their previous responses using a “back” button. |
| Response rates | Unique site visitor | We used IP addresses to ensure the uniqueness of responders. |
|  | View rate | We do not have enough data to estimate the view rate. |
|  | Participation rate | 702 participants completed the informed consent. 346 participants (49.29%) met the four inclusion criteria and started the survey.  249 |
|  | Completion rate | 279 out of 346 participants (80.64%) finished the survey. |
| Preventing multiple entries from the same individual | Cookies used | MTurk uses authentication cookies.  We eliminated duplicate entries (participants with same IP) before analysis by keeping the first entry. |
|  | IP check | We used participants’ IP addresses to identify the duplicate entries and eliminate them before analysis. |
|  | Log file analysis | We did not use other techniques to analyze the log file for identification of multiple entries. |
|  | Registration | Not applicable (this was an open survey). |
| Analysis | Handling of incomplete questionnaires | Only completed questionnaires were analyzed. |
|  | Questionnaires submitted with an atypical timestamp | We collected timestamps at the start and completion of the survey. Based on the number and length of the questions, we estimated the survey completion time 20 minutes. Responses with short completion times were excluded. We used a cut-off point of 5 minutes based on the time needed for a random click-through. |
|  | Statistical correction | No statistical correction was used in the analysis. |
